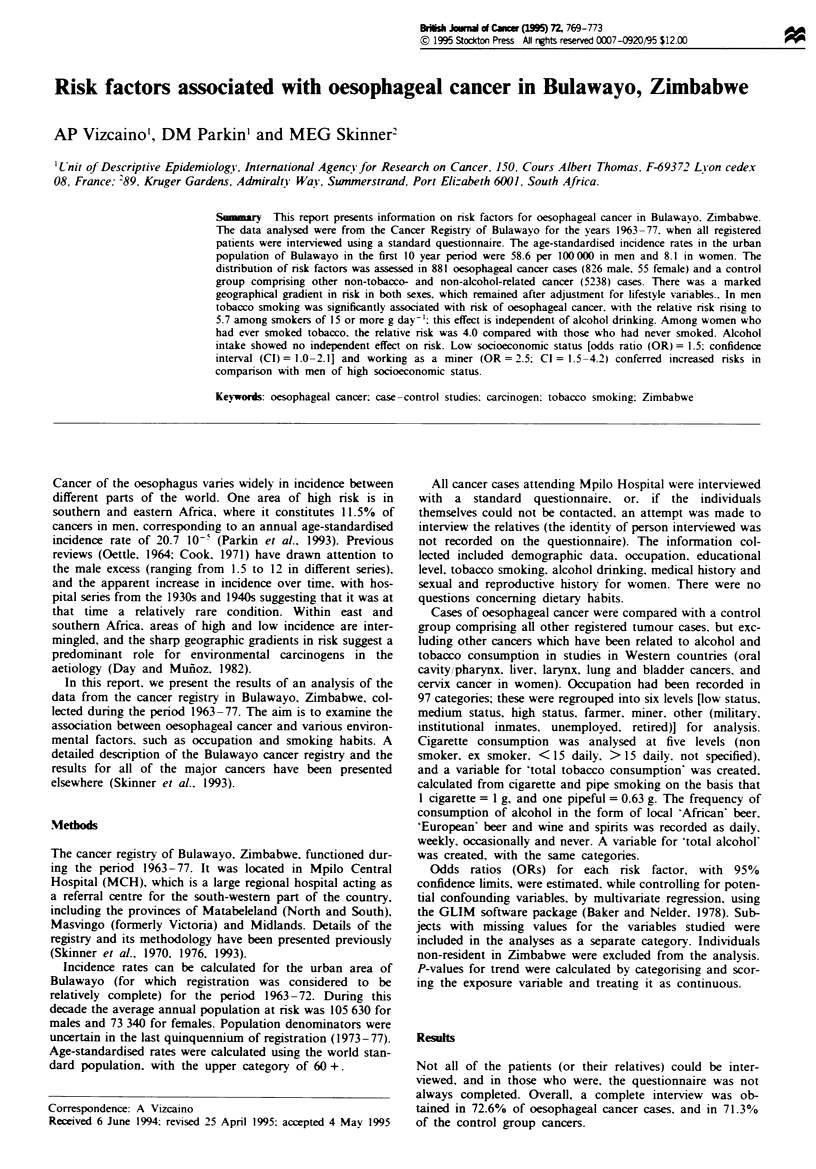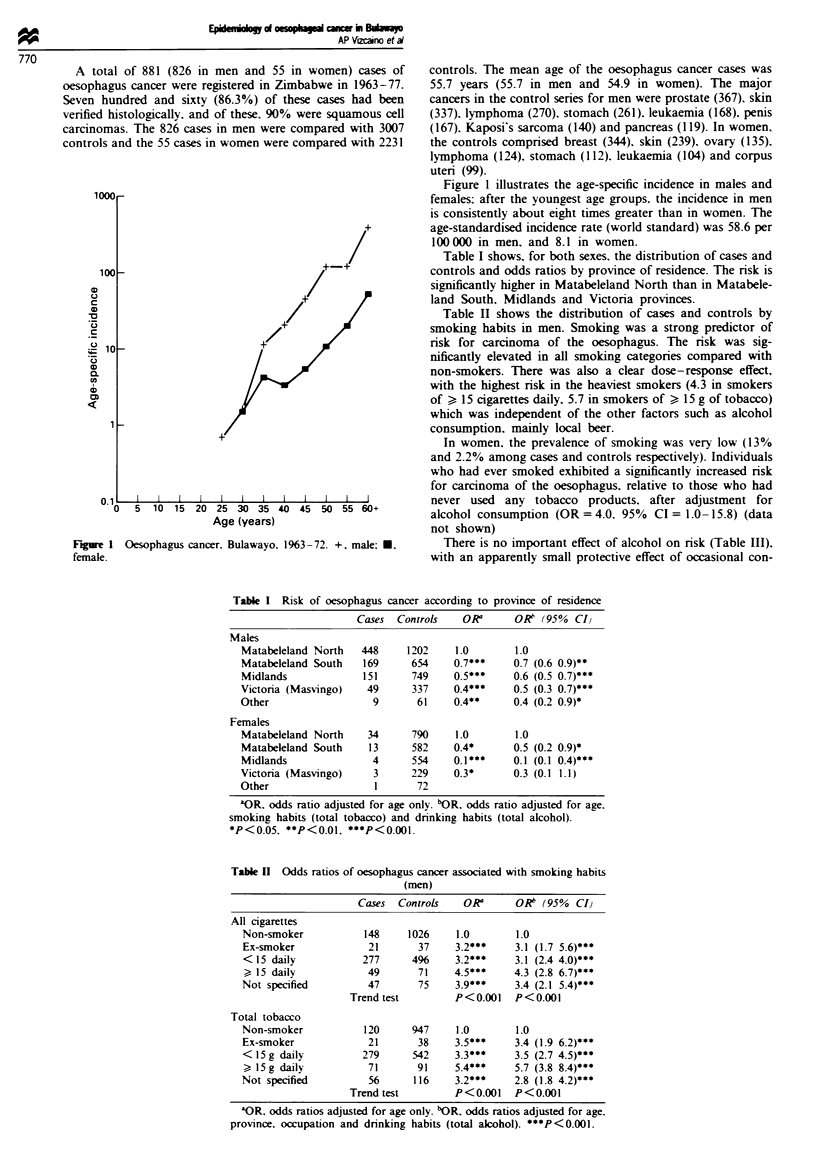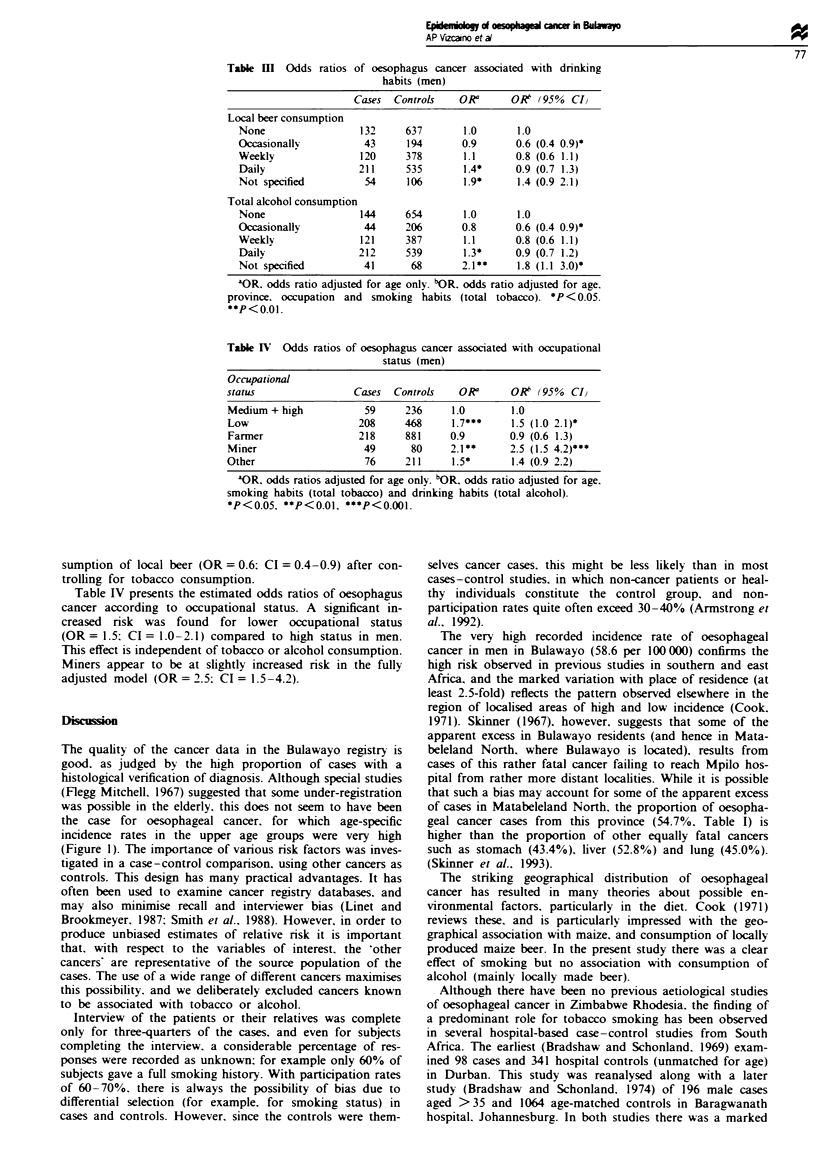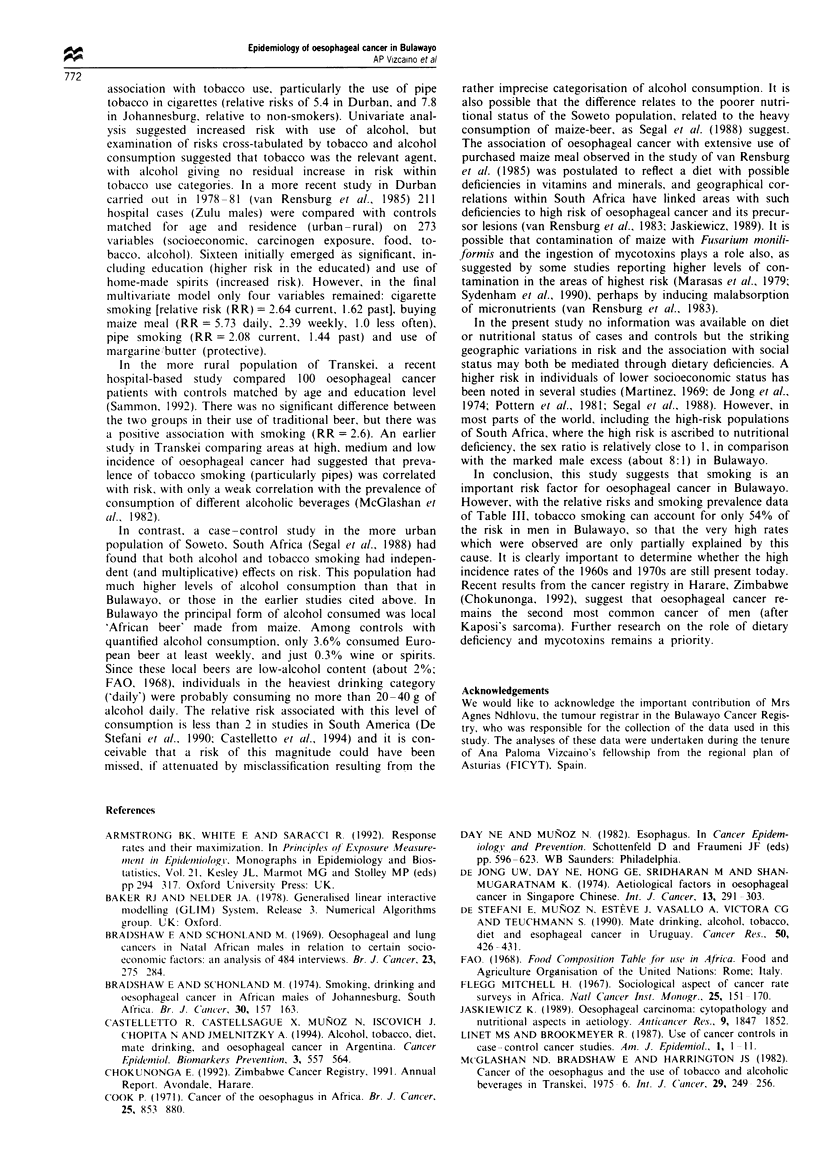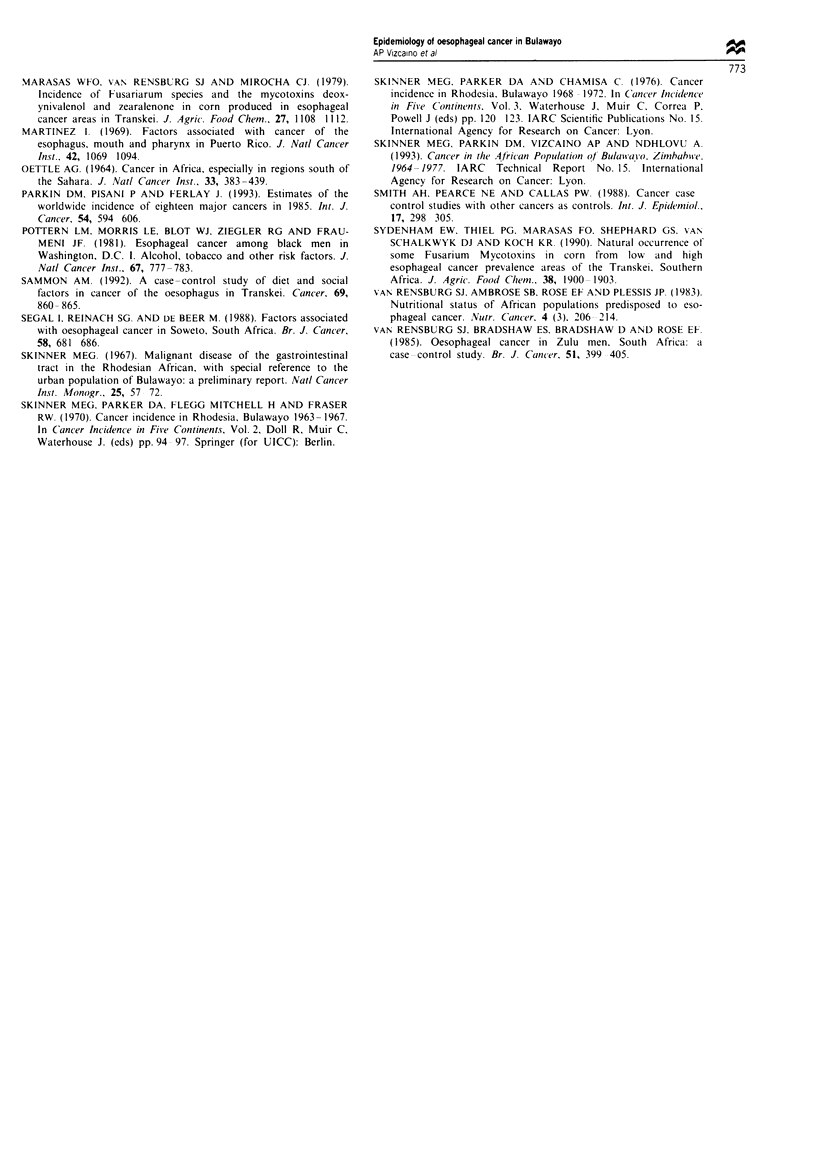# Risk factors associated with oesophageal cancer in Bulawayo, Zimbabwe.

**DOI:** 10.1038/bjc.1995.408

**Published:** 1995-09

**Authors:** A. P. Vizcaino, D. M. Parkin, M. E. Skinner

**Affiliations:** Unit of Descriptive Epidemiology, International Agency for Research on Cancer, Lyon, France.

## Abstract

This report presents information on risk factors for oesophageal cancer in Bulawayo, Zimbabwe. The data analysed were from the Cancer Registry of Bulawayo for the years 1963-77, when all registered patients were interviewed using a standard questionnaire. The age-standardised incidence rates in the urban population of Bulawayo in the first 10 year period were 58.6 per 100,000 in men and 8.1 in women. The distribution of risk factors was assessed in 881 oesophageal cancer cases (826 male, 55 female) and a control group comprising other non-tobacco- and non-alcohol-related cancer (5238) cases. There was a marked geographical gradient in risk in both sexes, which remained after adjustment for lifestyle variables. In men tobacco smoking was significantly associated with risk of oesophageal cancer, with the relative risk rising to 5.7 among smokers of 15 or more g day-1; this effect is independent of alcohol drinking. Among women who had ever smoked tobacco, the relative risk was 4.0 compared with those who had never smoked. Alcohol intake showed no independent effect on risk. Low socioeconomic status [odds ratio (OR) = 1.5; confidence interval (CI) = 1.0-2.1] and working as a miner (OR = 2.5; CI = 1.5-4.2) conferred increased risks in comparison with men of high socioeconomic status.


British Journal of Cancer (1995) 72, 769-773

© 1995 Stockton Press All rights reserved 0007-0920/95 $12.00

### Risk factors associated with oesophageal cancer in Bulawayo, Zimbabwe

AP Vizcaino[1], DM Parkin[1] and MEG Skinner[2]

[1]*Unit of Descriptive Epidemiology, International Agency for Research on Cancer, 150, Cours Albert Thomas, F-69372 Lyon cedex 08, France;* [2]*89, Kruger Gardens, Admiralty Way, Summerstrand, Port Elizabeth 6001, South Africa.*

**Summary** This report presents information on risk factors for oesophageal cancer in Bulawayo, Zimbabwe. The data analysed were from the Cancer Registry of Bulawayo for the years 1963-77, when all registered patients were interviewed using a standard questionnaire. The age-standardised incidence rates in the urban population of Bulawayo in the first 10 year period were 58.6 per 100 000 in men and 8.1 in women. The distribution of risk factors was assessed in 881 oesophageal cancer cases (826 male, 55 female) and a control group comprising other non-tobacco- and non-alcohol-related cancer (5238) cases. There was a marked geographical gradient in risk in both sexes, which remained after adjustment for lifestyle variables., In men tobacco smoking was significantly associated with risk of oesophageal cancer, with the relative risk rising to 5.7 among smokers of 15 or more g day$^{-1}$; this effect is independent of alcohol drinking. Among women who had ever smoked tobacco, the relative risk was 4.0 compared with those who had never smoked. Alcohol intake showed no independent effect on risk. Low socioeconomic status [odds ratio (OR) = 1.5; confidence interval (CI) = 1.0-2.1] and working as a miner (OR = 2.5; CI = 1.5-4.2) conferred increased risks in comparison with men of high socioeconomic status.

**Keywords**: oesophageal cancer; case-control studies; carcinogen; tobacco smoking; Zimbabwe

Cancer of the oesophagus varies widely in incidence between different parts of the world. One area of high risk is in southern and eastern Africa, where it constitutes 11.5% of cancers in men, corresponding to an annual age-standardised incidence rate of 20.7 $10^{-5}$ (Parkin *et al.*, 1993). Previous reviews (Oettle, 1964; Cook, 1971) have drawn attention to the male excess (ranging from 1.5 to 12 in different series), and the apparent increase in incidence over time, with hospital series from the 1930s and 1940s suggesting that it was at that time a relatively rare condition. Within east and southern Africa, areas of high and low incidence are intermingled, and the sharp geographic gradients in risk suggest a predominant role for environmental carcinogens in the aetiology (Day and Muñoz, 1982).

In this report, we present the results of an analysis of the data from the cancer registry in Bulawayo, Zimbabwe, collected during the period 1963-77. The aim is to examine the association between oesophageal cancer and various environmental factors, such as occupation and smoking habits. A detailed description of the Bulawayo cancer registry and the results for all of the major cancers have been presented elsewhere (Skinner *et al.*, 1993).

### Methods

The cancer registry of Bulawayo, Zimbabwe, functioned during the period 1963-77. It was located in Mpilo Central Hospital (MCH), which is a large regional hospital acting as a referral centre for the south-western part of the country, including the provinces of Matabeleland (North and South), Masvingo (formerly Victoria) and Midlands. Details of the registry and its methodology have been presented previously (Skinner *et al.*, 1970, 1976, 1993).

Incidence rates can be calculated for the urban area of Bulawayo (for which registration was considered to be relatively complete) for the period 1963-72. During this decade the average annual population at risk was 105 630 for males and 73 340 for females. Population denominators were uncertain in the last quinquennium of registration (1973-77). Age-standardised rates were calculated using the world standard population, with the upper category of 60+.

Correspondence: A Vizcaino
Received 6 June 1994; revised 25 April 1995; accepted 4 May 1995

All cancer cases attending Mpilo Hospital were interviewed with a standard questionnaire, or, if the individuals themselves could not be contacted, an attempt was made to interview the relatives (the identity of person interviewed was not recorded on the questionnaire). The information collected included demographic data, occupation, educational level, tobacco smoking, alcohol drinking, medical history and sexual and reproductive history for women. There were no questions concerning dietary habits.

Cases of oesophageal cancer were compared with a control group comprising all other registered tumour cases, but excluding other cancers which have been related to alcohol and tobacco consumption in studies in Western countries (oral cavity/pharynx, liver, larynx, lung and bladder cancers, and cervix cancer in women). Occupation had been recorded in 97 categories; these were regrouped into six levels [low status, medium status, high status, farmer, miner, other (military, institutional inmates, unemployed, retired)] for analysis. Cigarette consumption was analysed at five levels (non smoker, ex smoker, $<15$ daily, $>15$ daily, not specified), and a variable for 'total tobacco consumption' was created, calculated from cigarette and pipe smoking on the basis that 1 cigarette = 1 g, and one pipeful = 0.63 g. The frequency of consumption of alcohol in the form of local 'African' beer, 'European' beer and wine and spirits was recorded as daily, weekly, occasionally and never. A variable for 'total alcohol' was created, with the same categories.

Odds ratios (ORs) for each risk factor, with 95% confidence limits, were estimated, while controlling for potential confounding variables, by multivariate regression, using the GLIM software package (Baker and Nelder, 1978). Subjects with missing values for the variables studied were included in the analyses as a separate category. Individuals non-resident in Zimbabwe were excluded from the analysis. *P*-values for trend were calculated by categorising and scoring the exposure variable and treating it as continuous.

### Results

Not all of the patients (or their relatives) could be interviewed, and in those who were, the questionnaire was not always completed. Overall, a complete interview was obtained in 72.6% of oesophageal cancer cases, and in 71.3% of the control group cancers.

A total of 881 (826 in men and 55 in women) cases of oesophagus cancer were registered in Zimbabwe in 1963–77. Seven hundred and sixty (86.3%) of these cases had been verified histologically, and of these, 90% were squamous cell carcinomas. The 826 cases in men were compared with 3007 controls and the 55 cases in women were compared with 2231 controls. The mean age of the oesophagus cancer cases was 55.7 years (55.7 in men and 54.9 in women). The major cancers in the control series for men were prostate (367), skin (337), lymphoma (270), stomach (261), leukaemia (168), penis (167), Kaposi's sarcoma (140) and pancreas (119). In women, the controls comprised breast (344), skin (239), ovary (135), lymphoma (124), stomach (112), leukaemia (104) and corpus uteri (99).

**Figure 1** Oesophagus cancer, Bulawayo, 1963–72. +, male; ■, female.

Figure 1 illustrates the age-specific incidence in males and females; after the youngest age groups, the incidence in men is consistently about eight times greater than in women. The age-standardised incidence rate (world standard) was 58.6 per 100 000 in men, and 8.1 in women.

Table I shows, for both sexes, the distribution of cases and controls and odds ratios by province of residence. The risk is significantly higher in Matabeleland North than in Matabeleland South, Midlands and Victoria provinces.

Table II shows the distribution of cases and controls by smoking habits in men. Smoking was a strong predictor of risk for carcinoma of the oesophagus. The risk was significantly elevated in all smoking categories compared with non-smokers. There was also a clear dose–response effect, with the highest risk in the heaviest smokers (4.3 in smokers of ⩾ 15 cigarettes daily, 5.7 in smokers of ⩾ 15 g of tobacco) which was independent of the other factors such as alcohol consumption, mainly local beer.

In women, the prevalence of smoking was very low (13% and 2.2% among cases and controls respectively). Individuals who had ever smoked exhibited a significantly increased risk for carcinoma of the oesophagus, relative to those who had never used any tobacco products, after adjustment for alcohol consumption (OR = 4.0, 95% CI = 1.0–15.8) (data not shown)

There is no important effect of alcohol on risk (Table III), with an apparently small protective effect of occasional con-

**Table I** Risk of oesophagus cancer according to province of residence

| | *Cases* | *Controls* | *OR*[a] | *OR*[b] *(95% CI)* |
|---|---|---|---|---|
| Males | | | | |
| Matabeleland North | 448 | 1202 | 1.0 | 1.0 |
| Matabeleland South | 169 | 654 | 0.7*** | 0.7 (0.6 0.9)** |
| Midlands | 151 | 749 | 0.5*** | 0.6 (0.5 0.7)*** |
| Victoria (Masvingo) | 49 | 337 | 0.4*** | 0.5 (0.3 0.7)*** |
| Other | 9 | 61 | 0.4** | 0.4 (0.2 0.9)* |
| Females | | | | |
| Matabeleland North | 34 | 790 | 1.0 | 1.0 |
| Matabeleland South | 13 | 582 | 0.4* | 0.5 (0.2 0.9)* |
| Midlands | 4 | 554 | 0.1*** | 0.1 (0.1 0.4)*** |
| Victoria (Masvingo) | 3 | 229 | 0.3* | 0.3 (0.1 1.1) |
| Other | 1 | 72 | | |

[a]OR, odds ratio adjusted for age only. [b]OR, odds ratio adjusted for age, smoking habits (total tobacco) and drinking habits (total alcohol). *$P<0.05$, **$P<0.01$, ***$P<0.001$.

**Table II** Odds ratios of oesophagus cancer associated with smoking habits (men)

| | *Cases* | *Controls* | *OR*[a] | *OR*[b] *(95% CI)* |
|---|---|---|---|---|
| All cigarettes | | | | |
| Non-smoker | 148 | 1026 | 1.0 | 1.0 |
| Ex-smoker | 21 | 37 | 3.2*** | 3.1 (1.7 5.6)*** |
| < 15 daily | 277 | 496 | 3.2*** | 3.1 (2.4 4.0)*** |
| ⩾ 15 daily | 49 | 71 | 4.5*** | 4.3 (2.8 6.7)*** |
| Not specified | 47 | 75 | 3.9*** | 3.4 (2.1 5.4)*** |
| | Trend test | | $P<0.001$ | $P<0.001$ |
| Total tobacco | | | | |
| Non-smoker | 120 | 947 | 1.0 | 1.0 |
| Ex-smoker | 21 | 38 | 3.5*** | 3.4 (1.9 6.2)*** |
| < 15 g daily | 279 | 542 | 3.3*** | 3.5 (2.7 4.5)*** |
| ⩾ 15 g daily | 71 | 91 | 5.4*** | 5.7 (3.8 8.4)*** |
| Not specified | 56 | 116 | 3.2*** | 2.8 (1.8 4.2)*** |
| | Trend test | | $P<0.001$ | $P<0.001$ |

[a]OR, odds ratios adjusted for age only. [b]OR, odds ratios adjusted for age, province, occupation and drinking habits (total alcohol). ***$P<0.001$.

Table III Odds ratios of oesophagus cancer associated with drinking habits (men)

| | Cases | Controls | OR[a] | OR[b] (95% CI) |
|---|---|---|---|---|
| Local beer consumption | | | | |
| None | 132 | 637 | 1.0 | 1.0 |
| Occasionally | 43 | 194 | 0.9 | 0.6 (0.4 0.9)* |
| Weekly | 120 | 378 | 1.1 | 0.8 (0.6 1.1) |
| Daily | 211 | 535 | 1.4* | 0.9 (0.7 1.3) |
| Not specified | 54 | 106 | 1.9* | 1.4 (0.9 2.1) |
| Total alcohol consumption | | | | |
| None | 144 | 654 | 1.0 | 1.0 |
| Occasionally | 44 | 206 | 0.8 | 0.6 (0.4 0.9)* |
| Weekly | 121 | 387 | 1.1 | 0.8 (0.6 1.1) |
| Daily | 212 | 539 | 1.3* | 0.9 (0.7 1.2) |
| Not specified | 41 | 68 | 2.1** | 1.8 (1.1 3.0)* |

[a]OR, odds ratio adjusted for age only. [b]OR, odds ratio adjusted for age, province, occupation and smoking habits (total tobacco). *$P<0.05$. **$P<0.01$.

Table IV Odds ratios of oesophagus cancer associated with occupational status (men)

| Occupational status | Cases | Controls | OR[a] | OR[b] (95% CI) |
|---|---|---|---|---|
| Medium + high | 59 | 236 | 1.0 | 1.0 |
| Low | 208 | 468 | 1.7*** | 1.5 (1.0 2.1)* |
| Farmer | 218 | 881 | 0.9 | 0.9 (0.6 1.3) |
| Miner | 49 | 80 | 2.1** | 2.5 (1.5 4.2)*** |
| Other | 76 | 211 | 1.5* | 1.4 (0.9 2.2) |

[a]OR, odds ratios adjusted for age only. [b]OR, odds ratio adjusted for age, smoking habits (total tobacco) and drinking habits (total alcohol). *$P<0.05$, **$P<0.01$, ***$P<0.001$.

sumption of local beer (OR = 0.6; CI = 0.4–0.9) after controlling for tobacco consumption.

Table IV presents the estimated odds ratios of oesophagus cancer according to occupational status. A significant increased risk was found for lower occupational status (OR = 1.5; CI = 1.0–2.1) compared to high status in men. This effect is independent of tobacco or alcohol consumption. Miners appear to be at slightly increased risk in the fully adjusted model (OR = 2.5; CI = 1.5–4.2).

### Discussion

The quality of the cancer data in the Bulawayo registry is good, as judged by the high proportion of cases with a histological verification of diagnosis. Although special studies (Flegg Mitchell, 1967) suggested that some under-registration was possible in the elderly, this does not seem to have been the case for oesophageal cancer, for which age-specific incidence rates in the upper age groups were very high (Figure 1). The importance of various risk factors was investigated in a case–control comparison, using other cancers as controls. This design has many practical advantages. It has often been used to examine cancer registry databases, and may also minimise recall and interviewer bias (Linet and Brookmeyer, 1987; Smith *et al.*, 1988). However, in order to produce unbiased estimates of relative risk it is important that, with respect to the variables of interest, the 'other cancers' are representative of the source population of the cases. The use of a wide range of different cancers maximises this possibility, and we deliberately excluded cancers known to be associated with tobacco or alcohol.

Interview of the patients or their relatives was complete only for three-quarters of the cases, and even for subjects completing the interview, a considerable percentage of responses were recorded as unknown; for example only 60% of subjects gave a full smoking history. With participation rates of 60–70%, there is always the possibility of bias due to differential selection (for example, for smoking status) in cases and controls. However, since the controls were themselves cancer cases, this might be less likely than in most cases–control studies, in which non-cancer patients or healthy individuals constitute the control group, and non-participation rates quite often exceed 30–40% (Armstrong *et al.*, 1992).

The very high recorded incidence rate of oesophageal cancer in men in Bulawayo (58.6 per 100 000) confirms the high risk observed in previous studies in southern and east Africa, and the marked variation with place of residence (at least 2.5-fold) reflects the pattern observed elsewhere in the region of localised areas of high and low incidence (Cook, 1971). Skinner (1967), however, suggests that some of the apparent excess in Bulawayo residents (and hence in Matabeleland North, where Bulawayo is located), results from cases of this rather fatal cancer failing to reach Mpilo hospital from rather more distant localities. While it is possible that such a bias may account for some of the apparent excess of cases in Matabeleland North, the proportion of oesophageal cancer cases from this province (54.7%, Table I) is higher than the proportion of other equally fatal cancers such as stomach (43.4%), liver (52.8%) and lung (45.0%). (Skinner *et al.*, 1993).

The striking geographical distribution of oesophageal cancer has resulted in many theories about possible environmental factors, particularly in the diet. Cook (1971) reviews these, and is particularly impressed with the geographical association with maize, and consumption of locally produced maize beer. In the present study there was a clear effect of smoking but no association with consumption of alcohol (mainly locally made beer).

Although there have been no previous aetiological studies of oesophageal cancer in Zimbabwe/Rhodesia, the finding of a predominant role for tobacco smoking has been observed in several hospital-based case–control studies from South Africa. The earliest (Bradshaw and Schonland, 1969) examined 98 cases and 341 hospital controls (unmatched for age) in Durban. This study was reanalysed along with a later study (Bradshaw and Schonland, 1974) of 196 male cases aged >35 and 1064 age-matched controls in Baragwanath hospital, Johannesburg. In both studies there was a marked association with tobacco use, particularly the use of pipe tobacco in cigarettes (relative risks of 5.4 in Durban, and 7.8 in Johannesburg, relative to non-smokers). Univariate analysis suggested increased risk with use of alcohol, but examination of risks cross-tabulated by tobacco and alcohol consumption suggested that tobacco was the relevant agent, with alcohol giving no residual increase in risk within tobacco use categories. In a more recent study in Durban carried out in 1978–81 (van Rensburg *et al.*, 1985) 211 hospital cases (Zulu males) were compared with controls matched for age and residence (urban–rural) on 273 variables (socioeconomic, carcinogen exposure, food, tobacco, alcohol). Sixteen initially emerged as significant, including education (higher risk in the educated) and use of home-made spirits (increased risk). However, in the final multivariate model only four variables remained: cigarette smoking [relative risk (RR) = 2.64 current, 1.62 past], buying maize meal (RR = 5.73 daily, 2.39 weekly, 1.0 less often), pipe smoking (RR = 2.08 current, 1.44 past) and use of margarine/butter (protective).

In the more rural population of Transkei, a recent hospital-based study compared 100 oesophageal cancer patients with controls matched by age and education level (Sammon, 1992). There was no significant difference between the two groups in their use of traditional beer, but there was a positive association with smoking (RR = 2.6). An earlier study in Transkei comparing areas at high, medium and low incidence of oesophageal cancer had suggested that prevalence of tobacco smoking (particularly pipes) was correlated with risk, with only a weak correlation with the prevalence of consumption of different alcoholic beverages (McGlashan *et al.*, 1982).

In contrast, a case–control study in the more urban population of Soweto, South Africa (Segal *et al.*, 1988) had found that both alcohol and tobacco smoking had independent (and multiplicative) effects on risk. This population had much higher levels of alcohol consumption than that in Bulawayo, or those in the earlier studies cited above. In Bulawayo the principal form of alcohol consumed was local 'African beer' made from maize. Among controls with quantified alcohol consumption, only 3.6% consumed European beer at least weekly, and just 0.3% wine or spirits. Since these local beers are low-alcohol content (about 2%; FAO, 1968), individuals in the heaviest drinking category ('daily') were probably consuming no more than 20–40 g of alcohol daily. The relative risk associated with this level of consumption is less than 2 in studies in South America (De Stefani *et al.*, 1990; Castelletto *et al.*, 1994) and it is conceivable that a risk of this magnitude could have been missed, if attenuated by misclassification resulting from the rather imprecise categorisation of alcohol consumption. It is also possible that the difference relates to the poorer nutritional status of the Soweto population, related to the heavy consumption of maize-beer, as Segal *et al.* (1988) suggest. The association of oesophageal cancer with extensive use of purchased maize meal observed in the study of van Rensburg *et al.* (1985) was postulated to reflect a diet with possible deficiencies in vitamins and minerals, and geographical correlations within South Africa have linked areas with such deficiencies to high risk of oesophageal cancer and its precursor lesions (van Rensburg *et al.*, 1983; Jaskiewicz, 1989). It is possible that contamination of maize with *Fusarium moniliformis* and the ingestion of mycotoxins plays a role also, as suggested by some studies reporting higher levels of contamination in the areas of highest risk (Marasas *et al.*, 1979; Sydenham *et al.*, 1990), perhaps by inducing malabsorption of micronutrients (van Rensburg *et al.*, 1983).

In the present study no information was available on diet or nutritional status of cases and controls but the striking geographic variations in risk and the association with social status may both be mediated through dietary deficiencies. A higher risk in individuals of lower socioeconomic status has been noted in several studies (Martinez, 1969; de Jong *et al.*, 1974; Pottern *et al.*, 1981; Segal *et al.*, 1988). However, in most parts of the world, including the high-risk populations of South Africa, where the high risk is ascribed to nutritional deficiency, the sex ratio is relatively close to 1, in comparison with the marked male excess (about 8:1) in Bulawayo.

In conclusion, this study suggests that smoking is an important risk factor for oesophageal cancer in Bulawayo. However, with the relative risks and smoking prevalence data of Table III, tobacco smoking can account for only 54% of the risk in men in Bulawayo, so that the very high rates which were observed are only partially explained by this cause. It is clearly important to determine whether the high incidence rates of the 1960s and 1970s are still present today. Recent results from the cancer registry in Harare, Zimbabwe (Chokunonga, 1992), suggest that oesophageal cancer remains the second most common cancer of men (after Kaposi's sarcoma). Further research on the role of dietary deficiency and mycotoxins remains a priority.

**Acknowledgements**
We would like to acknowledge the important contribution of Mrs Agnes Ndhlovu, the tumour registrar in the Bulawayo Cancer Registry, who was responsible for the collection of the data used in this study. The analyses of these data were undertaken during the tenure of Ana Paloma Vizcaino's fellowship from the regional plan of Asturias (FICYT), Spain.

**References**

ARMSTRONG BK, WHITE E AND SARACCI R. (1992). Response rates and their maximization. In *Principles of Exposure Measurement in Epidemiology*, Monographs in Epidemiology and Biostatistics, Vol. 21, Kesley JL, Marmot MG and Stolley MP (eds) pp 294–317. Oxford University Press: UK.

BAKER RJ AND NELDER JA. (1978). Generalised linear interactive modelling (GLIM) System, Release 3. Numerical Algorithms group. UK: Oxford.

BRADSHAW E AND SCHONLAND M. (1969). Oesophageal and lung cancers in Natal African males in relation to certain socio-economic factors: an analysis of 484 interviews. *Br. J. Cancer*, **23**, 275–284.

BRADSHAW E AND SCHONLAND M. (1974). Smoking, drinking and oesophageal cancer in African males of Johannesburg, South Africa. *Br. J. Cancer*, **30**, 157–163.

CASTELLETTO R, CASTELLSAGUE X, MUÑOZ N, ISCOVICH J, CHOPITA N AND JMELNITZKY A. (1994). Alcohol, tobacco, diet, mate drinking, and oesophageal cancer in Argentina. *Cancer Epidemiol. Biomarkers Prevention*, **3**, 557–564.

CHOKUNONGA E. (1992). Zimbabwe Cancer Registry, 1991. Annual Report. Avondale, Harare.

COOK P. (1971). Cancer of the oesophagus in Africa. *Br. J. Cancer*, **25**, 853–880.

DAY NE AND MUÑOZ N. (1982). Esophagus. In *Cancer Epidemiology and Prevention*. Schottenfeld D and Fraumeni JF (eds) pp. 596–623. WB Saunders: Philadelphia.

DE JONG UW, DAY NE, HONG GE, SRIDHARAN M AND SHANMUGARATNAM K. (1974). Aetiological factors in oesophageal cancer in Singapore Chinese. *Int. J. Cancer*, **13**, 291–303.

DE STEFANI E, MUÑOZ N, ESTÈVE J, VASALLO A, VICTORA CG AND TEUCHMANN S. (1990). Mate drinking, alcohol, tobacco, diet and esophageal cancer in Uruguay. *Cancer Res.*, **50**, 426–431.

FAO. (1968). *Food Composition Table for use in Africa*. Food and Agriculture Organisation of the United Nations: Rome; Italy.

FLEGG MITCHELL H. (1967). Sociological aspect of cancer rate surveys in Africa. *Natl Cancer Inst. Monogr.*, **25**, 151–170.

JASKIEWICZ K. (1989). Oesophageal carcinoma: cytopathology and nutritional aspects in aetiology. *Anticancer Res.*, **9**, 1847–1852.

LINET MS AND BROOKMEYER R. (1987). Use of cancer controls in case–control cancer studies. *Am. J. Epidemiol.*, **1**, 1–11.

MCGLASHAN ND, BRADSHAW E AND HARRINGTON JS (1982). Cancer of the oesophagus and the use of tobacco and alcoholic beverages in Transkei, 1975–6. *Int. J. Cancer*, **29**, 249–256.

MARASAS WFO, VAN RENSBURG SJ AND MIROCHA CJ. (1979). Incidence of Fusariarum species and the mycotoxins deoxynivalenol and zearalenone in corn produced in esophageal cancer areas in Transkei. *J. Agric. Food Chem.*, **27,** 1108 1112.
MARTINEZ I. (1969). Factors associated with cancer of the esophagus, mouth and pharynx in Puerto Rico. *J. Natl Cancer Inst.*, **42,** 1069 1094.
OETTLE AG. (1964). Cancer in Africa, especially in regions south of the Sahara. *J. Natl Cancer Inst.*, **33,** 383–439.
PARKIN DM, PISANI P AND FERLAY J. (1993). Estimates of the worldwide incidence of eighteen major cancers in 1985. *Int. J. Cancer*, **54,** 594 606.
POTTERN LM, MORRIS LE, BLOT WJ, ZIEGLER RG AND FRAUMENI JF. (1981). Esophageal cancer among black men in Washington, D.C. I. Alcohol, tobacco and other risk factors. *J. Natl Cancer Inst.*, **67,** 777–783.
SAMMON AM. (1992). A case–control study of diet and social factors in cancer of the oesophagus in Transkei. *Cancer*, **69,** 860–865.
SEGAL I, REINACH SG AND DE BEER M. (1988). Factors associated with oesophageal cancer in Soweto, South Africa. *Br. J. Cancer*, **58,** 681 686.
SKINNER MEG. (1967). Malignant disease of the gastrointestinal tract in the Rhodesian African, with special reference to the urban population of Bulawayo: a preliminary report. *Natl Cancer Inst. Monogr.*, **25,** 57 72.
SKINNER MEG, PARKER DA, FLEGG MITCHELL H AND FRASER RW. (1970). Cancer incidence in Rhodesia, Bulawayo 1963–1967. In *Cancer Incidence in Five Continents*, Vol. 2, Doll R, Muir C, Waterhouse J. (eds) pp. 94 97. Springer (for UICC): Berlin.
SKINNER MEG, PARKER DA AND CHAMISA C. (1976). Cancer incidence in Rhodesia, Bulawayo 1968 1972. In *Cancer Incidence in Five Continents*, Vol. 3, Waterhouse J, Muir C, Correa P, Powell J (eds) pp. 120 123. IARC Scientific Publications No. 15. International Agency for Research on Cancer: Lyon.
SKINNER MEG, PARKIN DM, VIZCAINO AP AND NDHLOVU A. (1993). *Cancer in the African Population of Bulawayo, Zimbabwe, 1964–1977.* IARC Technical Report No. 15. International Agency for Research on Cancer: Lyon.
SMITH AH, PEARCE NE AND CALLAS PW. (1988). Cancer case control studies with other cancers as controls. *Int. J. Epidemiol.*, **17,** 298 305.
SYDENHAM EW, THIEL PG, MARASAS FO, SHEPHARD GS, VAN SCHALKWYK DJ AND KOCH KR. (1990). Natural occurrence of some Fusarium Mycotoxins in corn from low and high esophageal cancer prevalence areas of the Transkei, Southern Africa. *J. Agric. Food Chem.*, **38,** 1900 1903.
VAN RENSBURG SJ, AMBROSE SB, ROSE EF AND PLESSIS JP. (1983). Nutritional status of African populations predisposed to esophageal cancer. *Nutr. Cancer*, **4** (3), 206 214.
VAN RENSBURG SJ, BRADSHAW ES, BRADSHAW D AND ROSE EF. (1985). Oesophageal cancer in Zulu men, South Africa: a case control study. *Br. J. Cancer*, **51,** 399 405.